# Neural Kinesthetic Contribution to Motor Imagery of Body Parts: Tongue, Hands, and Feet

**DOI:** 10.3389/fnhum.2021.602723

**Published:** 2021-07-14

**Authors:** Irini Giannopulu, Haruo Mizutani

**Affiliations:** Interdisciplinary Centre for the Artificial Mind, Bond University, Gold Coast, QLD, Australia

**Keywords:** connectivity, kinesthetic representations, verbal actions, body parts, motor mental imagery

## Abstract

Motor imagery (MI) is assimilated to a perception-action process, which is mentally represented. Although several models suggest that MI, and its equivalent motor execution, engage very similar brain areas, the mechanisms underlying MI and their associated components are still under investigation today. Using 22 Ag/AgCl EEG electrodes, 19 healthy participants (nine males and 10 females) with an average age of 25.8 years old (sd = 3.5 years) were required to imagine moving several parts of their body (i.e., first-person perspective) one by one: left and right hand, tongue, and feet. Network connectivity analysis based on graph theory, together with a correlational analysis, were performed on the data. The findings suggest evidence for motor and somesthetic neural synchronization and underline the role of the parietofrontal network for the tongue imagery task only. At both unilateral and bilateral cortical levels, only the tongue imagery task appears to be associated with motor and somatosensory representations, that is, kinesthetic representations, which might contribute to verbal actions. As such, the present findings suggest the idea that imagined tongue movements, involving segmentary kinesthetic actions, could be the prerequisite of language.

## Introduction

Internal imagery, specifically mental imagery, is one of the most frequently used aspects of the mind as it allows for the simulation of sensations, motor, and/or verbal actions (Fox, 1914; Guillot et al., 2012; Bruno et al., 2018). The mental imagery preceding action accomplishment implies that actions need to be thought, prepared, planned, and organized before any execution takes place. Imagined actions, motor actions, in particular, occur on the basis of a specific cognitive process classically named “Motor imagery” (MI; Ehrsson et al., 2003). When used in research, participants are required to imagine executing an action without doing the corresponding movement (Papaxanthis et al., 2002; Fadiga et al., 2005). MI comprises the visualization of the required movement, which necessitates the implication of a dynamic brain network (Fadiga and Craighero, 2004; Li et al., 2018). Regardless of whether the movement is global, i.e., involving the whole body, or segmental, i.e., involving part of the body, the neural composition of this network is still under investigation. During MI, information associated with ongoing and preceding brain connections is activated (Friston, 2011). Multimodal in nature, this contains fundamental components of intra-structure synergy among motor, somatosensory, and visuospatial inputs (Naito et al., 2002; Vogt et al., 2013; Giannopulu, 2016, 2017, 2018). It has therefore been reported that during MI, people have similar kinesthetic (Jeannerod, 1994; Chholak et al., 2019) and/or tactile (Schmidt and Blankenburg, 2019) sensations to those they experience when effectively performing the movement that obviously involves motor and somesthetic functions (Frackoviak, 2004).

The “motor simulation hypothesis” suggests that MI associated with global or segmental body movements would enlist analogous motor representations (Fadiga and Craighero, 2004; Corballis, 2010). In accordance with this, some experimental studies support the idea of a temporal congruence between imagined and actual actions (Guillot et al., 2012). Some others suggest that the spatial distribution of neural activity during MI mirrors the spatial distribution of neural activity during actual movements (Miller et al., 2010). This latter includes important aspects to conceive the synchronization and coherence of neural activity between several brain areas. Neuroimaging studies in humans showed that when MI involves body parts and more particularly, hand movements, this typically initiates various areas associated with sensorimotor control areas including bilateral premotor, prefrontal supplementary motor, left posterior parietal area, and caudate nuclei (Gerardin et al., 2000). Recent brain network analysis has shown that frontal and parietal brain areas are also involved while performing MI of the right hand in third-person perspective (i.e., the subject was looking at a robotic hand movement) but not involved in the first-person perspective (i.e., the subject mentally controlled robot’s hand movement; Alanis-Espinosa and Gutiérrez, 2020). Moreover, in the prior mentioned study, prefrontal and sensorimotor brain areas demonstrated higher brain activity in first than in third-person perspective. Less contralateral and bilateral sensorimotor networks were observed for the left-hand MI, but no hemisphere lateralization for both left and right hands mental imagery was reported in the sensorimotor areas (Li et al., 2019). When right hand finger movements are considered, Binkofski et al. (2000) reported that they only involve Broca’s area. Based on the hypothesis that motor preparation shares the same mechanisms as MI (Jeannerod, 1994; Lotze and Halsband, 2006), Wang et al. (2020) analyzed the EEG patterns associated with the preparation of voluntary motion of left and right fingers and demonstrated that sensorimotor processes (essentially central C3 and C4 electrodes) were implicated. Open issues exist with regard to the neural involvement of the primary motor cortex in MI tasks, but studies have shown that the engagement of fingers, toes, and tongue leads to the activation of different subdivisions of the primary motor cortex together with the premotor cortex (Ehrsson et al., 2003). The premotor cortex and primary somatosensory area have demonstrated significant connections of unilateral left and right foot imagery movements in healthy participants (Gu et al., 2020). It was also proposed that regardless of the effector related to the observed movement, i.e., toe, finger, or tongue, the somatotopic organization reflecting the sensory homunculus would be engaged (Penfield and Baldrey, 1937; Penfield and Rasmussen, 1950; Stippich et al., 2002). Taken together, the above-mentioned data are consistent with the assumption that the MI associated with kinesthetic sensations affects somatotopic representations (Ehrsson et al., 2003).

The processes underlying motor action and verbal action are known to involve overlapping neural populations. For example, Broca’s area is not only implicated in language expression but is also activated during the execution of hand movements, oro-laryngeal, and oro-facial movements (Skipper et al., 2007; Clerget et al., 2009; Ferpozzi et al., 2018). Verbal information conveyed through hand gestures and oral sounds is processed in a similar way (Kohler et al., 2002; Fadiga et al., 2005). Nevertheless, mouth gestures seem to serve to disambiguate hand gestures (Emmorey et al., 2002). It was therefore, suggested that mouth actions, which are consistently associated with voicing and tongue actions, progressively granted authority over hand actions (Corballis, 2003). With that in mind, it seems that the evolutionary transition would be from hand to face to voice; in other words, from gesticulation to verbal generation, and was considered to be mediated by the mirror neuron system (MNS). This system becomes active during communicative mouth actions, i.e., lip-smacking (Ferrari et al., 2005), which are theorized to include somatosensory and motor patterns of the tongue and other areas within the mouth. In humans, moving a tongue is directly and unambiguously related to verbal actions expressed through complex sounds that take specific meanings when people communicate with one another (Corballis, 2010; Corballis, 2018).

In light of the aforementioned neuroanatomical and physiological arguments, it was hypothesized that tongue action imagery, which is widely associated with verbal actions, and obviously with the symbolic representations of these actions (Fadiga and Craighero, 2004; Rizzolatti and Sinigaglia, 2010), might cause stronger neural network activity and connectivity in the frontal areas than movements which involve upper (i.e., hands) and inferior (i.e., feet) limbs. To that end, network connectivity analysis in the topology of the whole brain was accomplished *via* graph theory (Beharelle and Small, 2016). Contrary to Qingsong et al. (2019) where the feature extraction of four-class motor signals of the same paradigm was performed on a general functional brain network, that is, without neural network specifications, the present study explores the implication of anterior (frontal) and posterior (parietal) brain networks on the tongue MI. Centrality [i.e., Degree centrality (DC) and betweenness centrality (BC)] and integration measures (i.e., local and global efficiency; Beharelle and Small, 2016) when healthy participants performed MI tasks involving different body parts, and more particularly, the tongue, left and right hand, and feet (Leeb et al., 2007; Bullmore and Sporns, 2009; Carlson and Millan, 2013), were considered. Expressly, it was hypothesized that the degree of centrality and betweenness centrality would be significantly higher in anterior frontal than in posterior parietal areas; that local and global efficiency would be significantly higher in frontal than in parietal areas for the MI of the tongue than for the other body parts.

## Materials and Methods

### Participants

Nineteen volunteers, nine males and 10 females participated in the study. The mean age was 25.8 years (s.d. 3.5 years). All were free from any known neurological disorder. All participants were right-handed. The experimental procedure was approved by the local ethics committee, the Bond University Human Research Ethics Committee (BUHREC 16065) and conformed to the National statement and the declaration of Helsinki 2.0. All subjects provided an informed consent.

### Procedure

**Figure 1:**
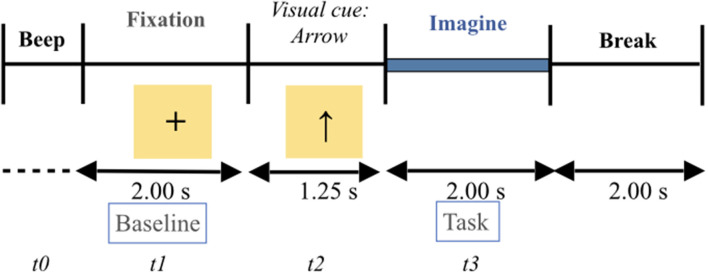
Timing presentation of the four motor imagery experimental paradigm. The start of each trial was signified by the presence of a short “beep tone”. This corresponds to *t* = 0. Immediately, a fixation cross appeared in the middle of the screen for 2 s, i.e., *t* = 1, fixation cross. The 2 s duration time constitutes the baseline. After these 2 s, an arrow indicating either left, right, up, or down direction appeared for 1.25 s on the screen. The arrow is the visual cue, i.e., *t* = 2. By definition, each direction of the arrow corresponds to a body part, as follows: left arrow **←** left hand (i.e., task 1), right arrow **→** right hand (i.e., task 2), up arrow **↑** tongue (i.e., task 3), and down arrow **↓** feet (i.e., task 4). Each arrow required the participant to perform the corresponding MI task. Once the arrow disappeared from the screen, each participant, facing the black screen, performed a MI task, one at a time, for 2 s, i.e., *t* = 3. At the end of each motor imagery task, each participant was allowed to take a break and relax. The inter-trial interval was around 2 s.

The participants were seated in an armchair in front of an LCD monitor. The experimental paradigm is a repetition of visual cue-based synchronous trials of four different MI tasks (e.g., Naeem et al., 2009; Tangermann et al., 2012; Friedrich et al., 2013). In accordance with this paradigm, all participants were instructed to imagine the movement of their body parts (i.e., first-person perspective) one by one: left hand (task 1), right hand (task 2), feet (task 3,) and tongue (task 4). A typical trial run is described in Figure 1. As presented in the Figure, the start of each trial was signified by the presence of a short “beep tone”. This corresponds to *t* = 0. Immediately, a fixation cross appeared in the middle of the screen for 2 s, i.e., *t* = 1, fixation cross. The 2 s duration time constitutes the baseline. After these 2 s, an arrow indicating either left, right, up, or down direction appeared for 1.25 s on the screen. The arrow was the visual cue, i.e., *t* = 2. By definition, each direction of the arrow corresponds to a body part, as follows: left arrow **←** left hand, right arrow **→** right hand, up arrow **↑** tongue, and down arrow **↓** feet. Each arrow required the participant to perform the corresponding MI task. Once the arrow disappeared from the screen, each participant, facing the black screen, performed a MI task, one at a time, for 2 s, i.e., *t* = 3. At the end of each MI task, each participant was allowed to take a break and relax. The inter-trial interval was around 2 s. The dataset of each participant consisted of two sessions. Each session comprised six sequences. Each of the four types of MI tasks (left hand, right hand, tongue, and feet) were displayed 12 times within each sequence in a randomized order, i.e., 72 trials per session. There were 288 trials in each session for each participant. The total number of trials was approximately 10,944.

The data analysis in this article compared the following periods of all the sequences of each session: fixation time (baseline), i.e., 2 s, with the MI task, i.e., 2 s.

### Data Recording

Twenty-two Ag/AgCl electrodes with an inter-electrode distance of 3.5 cm were used to record *via* the EEG. The electrode montage corresponds to the international 10–20 system: Fz, FC1, FC3, FCz, FC2, FC4, T7, C3, C1, Cz, C2, C4, T8, CP3, CP1, CPz, CP2, CP4, P3, Pz, P2, Oz. The signals were recorded monopolarly with the left mastoid serving as the reference and the right mastoid as ground. The signals were sampled with 250 Hz and bandpass-filtered between 0.5 Hz and 100 Hz. The sensitivity of the amplifier was set to 100 μV (Naeem et al., 2009). An additional 50 Hz notch filter was used to suppress line noise. Three more monopolar EOG (electrooculogram) channels were used and also sampled with 250 Hz. They were bandpass filtered between 0.5 Hz and 100 Hz with the 50 Hz notch filter enabled, and the sensitivity of the amplifier was set to 1 mv (Ang et al., 2012). The EOG channels were used for the subsequent application of artifact processing methods and were not be used for classification. A visual inspection of all datasets and trials containing artifacts was carried out by an expert. 4.8% of trials with EOG artifacts and 1.2% of trials with EMG (electromyographic) artifacts were marked and eliminated; while 94% of the trials were preserved. ICA algorithms decomposing the EEG signal into individual source signals were used.

### Graph Analysis

A simple graph (i.e., G = <V,E>) whose vertices (V) are unweighted, undirected, and multiples edges (E) was used. V (vertices) were represented by each of the 22 EEG electrodes (i.e., ch1 to ch22); E (edges) corresponded to each couple of electrodes as resulted by the adjacency matrix. The adjacency matrices for each body part to imagine (i.e., tongue, left hand, right hand, and feet) and the baseline were created with 22 rows and 22 columns (i.e., 22 vertices and 22 vertices) and cross-correlation coefficients were calculated for 22 × 22 combinations for each participant to assess the strength of the connection between the two electrodes (i.e., edges). To analyze the trends of whole brain topology network the properties of centrality and integration were considered. Centrality was represented by degree centrality and betweenness centrality. Integration was represented by local and global efficiency: (1) Degree centrality (DC) describes the number of vertices that are connected to a vertix. By definition, the more vertices, the more important the vertex is and similarly connected areas tend to communicate with each other; (2) betweenness centrality (BC) defines the part of all the shortest paths in the network that run through a given vertix. It facilitates functional integration. BC represents the vertix’s ability to make connections with other edges of the graph. The closer the vertices are to each other, the shorter is the path length and the more efficient is the transfer of the information between them, and (3) efficiency (local and global) (E) measures topological distances between vertices and how accurately the vertices communicate between them. Local efficiency reflects interconnected neighboring vertices and global efficiency indices the interconnectedness of all vertices across the entire brain.

### Data Analysis

The adjacency matrices for each MI task and the baseline were considered and the produced functional connectivity values were transformed into z-scores (i.e., normalization with zero mean and variance three separately for each participant). In that way, each element of each correlation matrix corresponded to the *z* scores for each vertices combination (Rubinov and Sporns, 2010). In addition, a permuted *t*-test was computed to assess the differences between each imagery task (i.e., left hand, right hand, feet, and tongue) and the baseline. To regulate the statistical confidence measures (i.e., the resulted values; Genovese et al., 2002; Rouam, 2013), the False Discovery Rate (FDR) approach was then applied and *q*-values (i.e., adjusted p-values) were implemented to determine the resulted differences as an alternative to the *p*-value. Such an approach allowed the incorrectly rejected null hypotheses, i.e., type I error, to be determined among all significant results (Benjamini and Hochberg, 1995; Schwartzman and Lin, 1999).

Low-Resolution Brain Electromagnetic Tomography (LORETA) transform was used to perform source analysis using BESA^®^ Research 7.0 software (BESA^®^) to identify which brain areas were activated during the baseline and the MI task. LORETA was used to adjust brain areas in 3D space. Time windows were selected in 50 ms at the time of 2 s (baseline) and 2 s (imagery task) from the beginning of the epoch. The scale was set between 0–400 × 10^−3^ nAm/cm^3^.

The cortical network across the sources, i.e., global EEG coherence across all frequency bands (1–45 Hz), a functional connectivity analysis was performed by applying the coherence method (Rosenberg et al., 1989; Laptinskaya et al., 2020) followed by complex demodulation with time and frequency sampling 0.5 Hz, 100 ms in BESA Connectivity 1.0 software (BESA^®^). The number of signal sources for the connectivity analysis was selected as 10, and the locations of dipole orientations in the brain were calculated using the genetic algorithm. The connectivity between these sources was displayed in 3D brain mode using the LORETA software.

## Results

**Figure 2:**
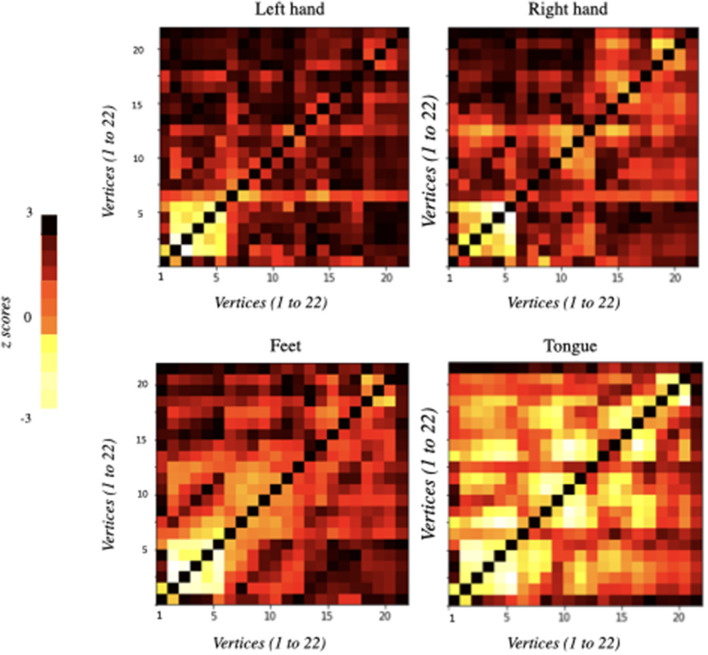
Functional connectivity matrices obtained from the comparison of EEG data between baseline and imagery tasks. They were ordered according to the motor imagery task: left hand, right hand, feet, and tongue. Information flows from the vertices marked on the vertical axis to the vertices marked on the horizontal axis: 22 vertices × 22 vertices (i.e., 22 electrodes × 22 electrodes). Each element of the correlation matrix shows z-scores corresponding to each node comparison. Statistically significant results were only obtained for tongue motor imagery (*p* < 0.05).

The functional connectivity matrices resulting from the differences between the baseline and each MI task separately (i.e., left hand, right hand, feet, and tongue) are represented in Figure 2. The only statistically significant results were observed for the tongue imagery task. All vertices correlations in the remaining imaginary tasks were not statistically significant. Only the statistically significant results will be considered in the following analysis.

Table 1 displays the largest 19 vertices correlations that were significantly reduced during tongue imagery movement compared to the baseline (adjusted *p*-values are described above). The 19 statistically significant combinations in the tongue imagery task include seven in a left hemisphere, two in a right hemisphere, and 10 over both hemispheres.

**Table 1:** Presentation of both inter-hemispheric and intra-hemispheric coherence combinations between 22 × 22 vertices and adjusted *p*-values (i.e., *q*-values) after False Discovery Rate (FDR) computation. The top 19 vertices statistically significant combinations in which the reduction appeared during the four-task experimental paradigm are associated with the tongue motor imagery only.

Electrode montage	Coherence pair	Adjusted *p*-value after FDR
*Left hemisphere*
ch3–ch8	FC1-C3	1.87e-06
ch9–ch10	C1-Cz	1.76e-05
ch2–ch3	FC1-FC3	8.76e-06
ch9–ch14	C1-CP3	4.32e-06
ch8–ch9	C3-C1	3.33e-05
ch8–ch10	C3-Cz	3.33e-05
ch3–ch10	FC3-Cz	3.02e-05
*Right hemisphere*
ch5–ch6	FC2-FC4	6.98e-05
ch6–ch13	FC4-T8	4.03e-05
*Both Hemisphere*
ch3–ch4	FC1-FCz	7.68e-05
ch4–ch8	FC2-C3	2.73e-06
ch3–ch11	FC1-C2	5.37e-06
ch4–ch9	FCz-C1	5.01e-05
ch10–ch11	Cz-C2	1.87e-06
ch4–ch12	FCz-C4	3.42e-05
ch1–ch12	Fz-C4	8.43e-06
ch6–ch10	FC4-Cz	3.86e-05
ch5–ch8	FC2-C3	7.10e-06
ch5–ch7	FC2-T7	5.24e-05

**Figure 3:**
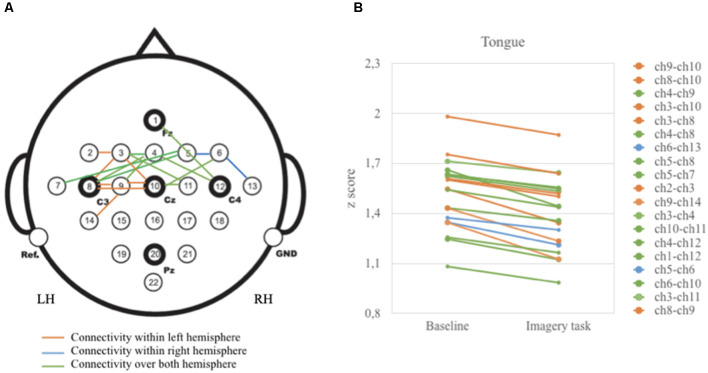
Graphical presentation on the relationship between baseline and tongue imagery task **(A)** 19 vertices significant correlations **(B)** configuration of z-scores. Brain activity is reduced when participants performed the tongue imaginative task. Note that vertices correlations were statistically significant for the anterior and central brain areas (*p* < 0.05).

As illustrated in Figure 3, vertices correlations in the left hemisphere and over both left and right hemispheres displayed relatively higher *z*-scores than in the right hemisphere. These correlations are highly present in the anterior (i.e., frontal) and central (i.e., frontal and parietal) areas.

Three key parameters representing network properties (Rubinov and Sporns, 2010), such as measures of centrality and integration, were computed. Measures of centrality were typified by degree centrality (DC) and betweenness centrality (BC). The average and the standard deviation of degree centrality were 3.87 ± 0.872 respectively with the highest number of vertices (5) in FC3, C3, Cz, followed by FCz, C1 (4 vertices), and FC2 and FC4 (3 vertices). In other words, the degree of centrality was higher in anterior (frontal) and central (frontoparietal) than posterior brain areas. Betweenness centrality (BC) measures gave the highest index for anterior (frontal) and central (frontoparietal) areas for the MI of the tongue (*t* = 13.32, *p* < 0.05, Cohen’s *d* = 0.78) but not for the MI of the other body parts (i.e., *t* = 0.65, *p* > 0.05 for the left hand; *t* = 1.25, *p* > 0.05 for the right hand; *t* = 1.02, *p* > 0.05 for the feet).

Measures of integration were represented by both local and global efficiency (E). Local efficiency is associated with the features of how interconnected neighboring vertices are to each other. The average and standard deviation of local efficiency was constantly higher during the rest condition (mean = 2.154, sd = 0.054) compared to the imagined condition for the tongue (mean = 1.646, sd: 0.032; *t* = 12.94, *p* < 0.05, Cohen’s *d* = 0.69). However, local efficiency was not significantly different across the other imagined conditions (i.e., *t* = 0.52, *p* > 0.05 for the left hand; *t* = 0.88, *p* > 0.05 for the right hand; *t* = 0.23, *p* > 0.05 for the feet). Similarly, the average and standard deviation of global efficiency, that is, the measure of the interconnectedness of all vertices across the entire brain, was significantly higher during the rest condition (mean = 1.323, sd = 0.021) compared to the tongue imagery condition (mean = 0.634, sd = 0.015; *t* = 13.22, *p* < 0.05, Cohen’s *d* = 0.71) but not compared to all the other MI conditions (i.e., *t* = 0.22, *p* > 0.05 for the left hand; *t* = 2.15, *p* > 0.05 for the right hand; *t* = 0.99, *p* > 0.05 for the feet).

In order to further verify the validity of the above results, the topology of the electrical activity in the brain based on multichannel surface EEG (i.e., LORETA) and brain maps of coherence connectivity were computed and described. The direct comparison of the four body parts (i.e., left hand, right hand, tongue, and feet) was also reported.

**Figure 4:**
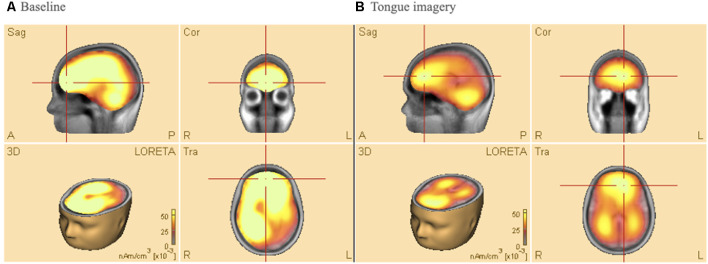
Low-resolution electromagnetic tomography (LORETA) source analysis during **(A)** baseline **(B)** tongue imagery. On average, the mirrored intensity of the brain activity was reduced for the tongue imagery in premotor, motor, parietal and visual areas bilaterally (*p* < 0.01). The orange/yellow scale is a positive one, i.e., the more high, the more intense. Orange/Yellow shades indicate decreased sources. Abbreviations: L: left; R: right; A: anterior; P: posterior; Sag: Sagittal; Cor: Coronal.

Figure 4 shows average results of Low-Resolution Electromagnetic Tomography (LORETA) source analysis in both baseline (a), and imagery task (b) conditions when the participants performed the tongue MI. The data of all participants were analyzed to find 3D determination with the intensity of brain activity in those conditions. The estimated LORETA images mirror the intensity of the brain activity, which tended to reduce during the tongue MI in anterior (premotor and motor areas) and posterior (parietal and visual) areas of both hemispheres (*t* = 13.67, *p* < 0.01, Cohen’s *d* = 0.75 and *t* = 18.7 *p* < 0.01, Cohen’s *d* = 0.68 respectively).

**Figure 5:**
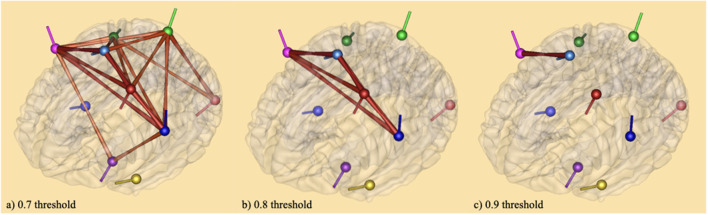
Brain maps of coherence connectivity at **(A)** 0.7 threshold **(B)** 0.8 threshold and **(C)** 0.9 threshold in relation to tongue imagery. A coherence method followed by complex demodulation with time and frequency sampling 100 ms and 0.5 Hz respectively was applied in BESA Connectivity 1.0 software (BESA^®^). When the 0.7 threshold is considered, the connections across the vertices were significant for the baseline vs. tongue imagery (*p* < 0.05) only. The vertices represent the significant brain areas reported by the source space analysis. The edges are loaded and colored according to the connectivity strength. They indicate significant higher (intense color) and lower (soft color) areas of synchronization both bilaterally and unilaterally.

The results of connectivity analysis using coherence described in the method section are given in Figure 5. The coherence method was applied to average source analyses with 19 individual subjects. This method gives the functional connectivity of different brain areas, that is, the synchronicity of brain activities at both bilateral and unilateral levels (Friston, 2011). It was followed by complex demodulation with time and frequency sampling 100 ms and 0.5 Hz applied respectively in BESA Connectivity 1.0 software (BESA^®^). Thresholding was based on the following observations: (1) thresholds can be defined arbitrarily (Garrison et al., 2014); (2) a narrow range of thresholds (*r* = 0.05–0.4 or *r* = 0.03–0.5) can lead to incomplete or misleading results (van den Heuvel et al., 2009; Formito et al., 2010); (3) thresholds can be applied based on a correlation coefficient of *r* = 0.1–0.8 (Buckner et al., 2009; Tomasi and Volkow, 2010). In attempts to avoid inaccurate results, the connections between the sources on the brain areas were illustrated with three thresholds: 0.7 (a), 0.8 (b), and 0.9 (c). At 0.7 threshold (a), the connections crosswise the vertices were significant for the baseline vs. tongue imagery task. The vertices represent the significant areas revealed by the source space analysis; the edges are graded and colored according to their connectivity strength. The edges connecting the sources indicate the cortical connections. The networks were significant at *p* < 0.05. Some of the strongest connectivities are depicted in the frontal (i.e., premotor, motor) and somatosensory cortical areas (i.e., kinesthetic areas) for the entire tongue imagery task with reference to the baseline. These areas also appear to be connected with motor (frontal) related areas in both hemispheres which appear to be strongly associated with tongue imagery.

A one-way Anova was conducted to test for differences in neural activity during MI of the body parts (left and right hand, feet, and tongue). In completing the imagery tasks, participants’ neural activity was significantly affected, *F*_(2,18)_ = 6.638, *p* < 0.05, η^2^ = 0.1579. Tukey *post hoc* comparisons showed that tongue mental imagery (*M* = 2.65, sd = 0.54) involves greater neural activity than the mental imagery of the left (*M* = 1.29, sd = 0.99), and right (*M* = 1.01, sd = 0.67) hand and the feet (*M* = 1.38, sd = 0.77).

## Discussion

In the present study, brain activity was analyzed when healthy adults were required to imagine performing voluntary movements of their body parts: left and right hands, feet, and the tongue. Scrutinizing interactions between regions on a whole-brain level, and identifying the role of individual regions within a network, graph theory method, and coherence approach were used (Beharelle and Small, 2016). Consistent with the hypothesis, the results revealed significant neural implications when participants imagined complete tongue movements only. The present findings might reflect a complex network connected to the left hemisphere and to both hemispheres. More precisely, the brain areas associated with tongue imagery, appear to involve the premotor cortex of the left hemisphere (unilaterally), the primary motor cortex, i.e., M1 and the primary somatosensory area (S1; bilaterally). Interestingly, and regardless of the type of tongue movement, these results are coherent with existing data (Ehrsson et al., 2003) reporting that tongue MI appears to preferentially engage the edge areas, and likely 4a and 4p. Both these areas have extensive cortico-cortical projections from higher-order motor regions, specifically with Broca’s area (Buccino et al., 2004), and receive inputs from somesthetic areas (Geyer et al., 1999). During tongue MI, the premotor cortex seems to be activated too. The involvement of this cortical region is consistent with the fact that area 6 is considered to be active and plays a role in planning movement, in using abstract rules to achieve tasks, and in verbal production. From a neuroanatomical point of view, the premotor cortex (area 6) could be seen to have distinct interconnectivity to the primary motor cortex (area 4) and this latter is interconnected to the ascendant parietal cortex, i.e., the primary somatosensory cortex (1, 2, 3), as well as to the superior (5, 7) and inferior (39, 40) parietal cortices. This suggests that the motor and somatosensory activations observed during the imagery task, i.e., both premotor and primary motor areas, might be interconnected and can be assumed to depend on top-down influences from other motor areas of the frontal cortex (Lissek et al., 2013) including Broca’s area in the left and the right hemispheres.

Consistent with previous studies (Ehrsson et al., 2003; Kilteni et al., 2018), the current findings imply that imagery of tongue movement might involve somatopically organized parts of the frontal and parietal cortices bilaterally. In other words, tongue imagery would engage the motor and somatosensory areas, that is, kinesthetic areas (Jacobs, 2011). Altogether, the findings support the idea that in healthy participants the brain would construct internal kinesthetic simulations of tongue movements. The patterns of motor activation during mental imagery observed in the present study are not only consistent with existing studies, but also provide support for the simulation hypothesis of MI (Papaxanthis et al., 2002; Fadiga and Craighero, 2004; Corballis, 2010; Piedimonte et al., 2014). Following this hypothesis, the motor representations engaged when an action is executed are also present when the same action is imagined. In the present situation, this signifies that internal imagery of specific body parts, i.e., the tongue, activates analogous somesthetic representations in addition to motor representations. When qualified movements are achieved, such as the ones that have been used in this study, the somatosensory cortex, and more specifically, the primary somatosensory cortex, has a key role in processing afferent somatosensory inputs. The somatosensory cortex appears to support the integration of both sensory and motor signals on the basis of co-representations (Borich et al., 2015). Studies have revealed a relationship between the type of stimulation, i.e., visual stimulation that the participants have imagined, and the activation of the corresponding brain areas, i.e., the visual brain areas (O’Craven and Kanwisher, 2000). Currently, even if a visual cue preceded the imagery task, motor and somatosensory activations, i.e., kinesthetic activations, visual activations were not significantly correlated with the imaginative task. Taken together, the present findings and the above-mentioned interconnections between frontal and parietal regions, build on the existing observations related to both motor and somatosensory areas. Accordingly, this suggests that a relationship exists between the movement that has been imagined and the activation patterns of somatotopically organized motor and somesthetic areas, i.e., kinesthetic areas. Specifically, the tongue imagery task would be associated with both motor and somatosensory representations, namely kinesthetic representations not only in the left but also in the right hemisphere.

Contrary to studies reported on somatotopic unilateral or bilateral forward (i.e., prefrontal and/or frontal) and backward (i.e., parietal) activations when participants were instructed to perform MI tasks of the right or left hand (Li et al., 2019; Alanis-Espinosa and Gutiérrez, 2020) and the left or right foot (Gu et al., 2020), the current investigation did not report somatotopic motor and/or somesthetic activations, i.e., motor and/or somesthetic representations of the upper and lower limbs. The inconsistencies between our results and the above-mentioned findings could be understood from a methodological point of view. First, the sample size importantly differs between the studies: only two subjects performed the motor hand imagery task *via* brain-computer interfaces (BCI) in the investigation of Alanis-Espinosa and Gutiérrez (2020), and 10 participants performed the feet imagery task in the Gu et al. (2020). In the study of Li et al. (2019), a conventional number of 40 participants was initially included but only the data of 22 subjects, totally unbalanced between male (21 subjects) and female (one subject), were analyzed. Although limited, the sample of our study was balanced as it was composed of nine males and 10 females. Second, the studies differ with regard to their objectives. For instance, the aim of the Alanis-Espinosa and Gutiérrez (2020) study was to control the movement of a robotic hand using MI at the first and third-person perspective. For the other studies, the aim was to imagine moving different body parts according to a defined experimental design using various visual stimuli (Li et al., 2019; Gu et al., 2020). Also, a specific MI paradigm was included in each study. More particularly, BCI was utilized to control an immersive telepresence system *via* MI (Alanis-Espinosa and Gutiérrez, 2020), but MI of the upper limbs (Li et al., 2019) and the lower limbs (Gu et al., 2020) was also performed. In the present study, a totally different procedure was employed where participants were instructed to complete a 4-class imagery task of the tongue and the upper and lower body limbs. Next, even if graph theory was employed in all the aforementioned studies, each study included specific graph-theoretical parameters on EEG data analysis, which were associated with predefined (or not) ROI (regions of interest) and statistical analyses depending on the proffered hypotheses. Some studies analyzed event-related desynchronization/synchronization (ERD/ERS) on specific electrodes (Li et al., 2019) while others investigated alpha and beta oscillation networks (Gu et al., 2020) or theta, alpha, beta, and gamma oscillations (Alanis-Espinosa and Gutiérrez, 2020) associated with predefined ROIs. Lastly, as all the above results are dependant on the selected neuroimaging techniques, the obtained observations might reflect and be understood by the limited spatial resolution of EEG. Expressly, in the current study, 22 Ag/AgCl electrodes were used. In contrast, 15 Ag/AgCl and 32 Ag/AgCI electrodes were managed by Li et al. (2019) and Alanis-Espinosa and Gutiérrez (2020) respectively, and 64 Ag/AgCl electrodes were employed by Gu et al. (2020). Note that the electrode montage differs with regard to the international electrode system (i.e., 10–10 vs. 10–20), the correspondent electrode impedance, EEG data digitalization, and bandpass filter. Moreover, the differences between the present results and the aforementioned studies can also be explained by the impossibility to obtain the real source signal (Gu et al., 2020), and also by the fact that the physiological correlates of actual, not imagined movements, are not always precisely organized (Ehrsson et al., 2003). Finally, given the temporal resolution of the EEG technique, it is also likely that the time it takes to imagine a specific movement depends on the procedure that has been used and the instructions that have been given to the participants. In this study, a classic 4-class imagery paradigm was used (e.g., Naeem et al., 2009; Tangermann et al., 2012; Friedrich et al., 2013) where the participants were invited to mentally simulate the movements of different body parts without receiving explicit instructions, i.e., what kind of movement the participants should and should not imagine. One could expect that the absence of explicit instructions would facilitate the activation of cortical areas due to electromyographic EMG activity (Bruno et al., 2018). On the contrary, the current findings support the idea that motor activation associated with muscle activity does not occur during “non pure” MI tasks, even when explicit instructions to avoid overt movements are not given to the participants. With that in mind, these findings are consistent with studies demonstrating that patterns of activation, which are very close at cortical level, as in the case for the feet imagery motor task, cannot really be discriminated (Penfield and Baldrey, 1937; Penfield and Rasmussen, 1950; Graimann et al., 2010). Additionally, the presence of motor and somesthetic activations during the tongue imagery task and their absence during the hand imagery tasks is also consistent with the assumption that tongue actions gradually overshadow hand actions (Corballis, 2003).

In the present study, all participants were untrained and instructed to imagine the movement of their body parts: right and left hand, feet, and tongue. Due to the data which states that motor commands for muscle contractions are blocked by the motor system *via* inhibitory processes even when prepared during the imagery task (Jeannerod, 1994; Roosink and Zijdewind, 2010; Guillot et al., 2012), the EMG activity was not recorded during the imaginative tasks. Instead, all trials containing EMG and EOG artifacts were marked and eliminated after visual inspection made by an expert. The presence of cortical activity during the tongue MI and its absence during hand and feet imagery, simply indicates that there is no reason to speculate that the obtained findings refer to actual muscle contraction instead of an imaginative task. In addition, in the current situation, the tongue imagery task that participants performed was a voluntary active mental imagery of common movements: the movements people perform when they verbally communicate with other people. Interestingly, similar results were reported even when participants were instructed to perform specific tongue movements during which electromyographic activity was recorded (Ehrsson et al., 2003). Note that tongue movements are verbal and nonverbal, i.e., onomatopoeias, movements *per se*. The participants were invited to a conscious mental action, that is, to mentally manipulate an internal representation of their own body parts, i.e., a kinesthetic representation. Recent studies theorized that the mental imagery of the upper limbs performing motor actions depends on the intensity of their “ownership” (Alimardani et al., 2013, 2014). Taken together, the above and actual findings suggest that the ownership hypothesis could concern both nonverbal motor actions and verbal actions.

Limitations of the study are partly due to its exploratory nature and methodological choices. Although the obtained results are statistically significant, it is acknowledged that the sample size of the current study is limited to 19 participants. To further validate the results, it is planned to increase sample size and also include neurological patients (e.g., frontal and parietal diseases) to perform intra and inter individual comparisons. A classic 4-class experimental paradigm (left, right hand, feet, and tongue) was utilized and untrained healthy participants were tested. This paradigm is commonly used, and several studies have been published (e.g., Naeem et al., 2009; Tangermann et al., 2012; Friedrich et al., 2013), but it is agreed that a new topology has to be introduced in order to improve it. The paradigm can be improved by the introduction of new cues. For instance, in the present study, visual cues were utilized (i.e., four arrows) and participants were instructed to imagine their body parts one by one (i.e., each arrow corresponds to a body part). It would be interesting to facilitate participants’ insight by introducing direct visual (i.e., the image of the body part to imagine) and/or somesthetic cues (i.e., touching each body part to imagine) into the paradigm. Moreover, when body parts are taken into account, the left and the right foot should be included in a future study. This is not the case in the present study. The current research proposes new paradigms for maximizing neural network recruitment, but these paradigms depend on the limited spatial resolution of the 22-EEG system that has been used. In a future study, a 62-EEG system and additional graph theory parameters for a deeper analysis of the data should be employed. Finally, and given the results, it is obvious that the speculated relationship between the imagined tongue movements and language (both expression and comprehension) needs to be tested on a new methodological ground.

In conclusion, using the internal perspective essentially associated with visual and kinesthetic information, the results of the present study support the idea that only one movement, the movement of the tongue, which is a skilled movement in itself, was imagined using the motor and the somesthetic mechanisms from a first-person view (i.e the imagined speaker). This makes particular sense when considering that the tongue and the mouth are both structurally and conceptually connected to verbal communication (Corballis, 2003, 2010; Buccino et al., 2004; Gallese, 2005; Xu et al., 2009). It implies that even if the participants were not explicitly instructed to imagine a specific tongue movement, tongue movements are specific *per se* and are directly and/or indirectly related to verbal actions. It also implies that the neural architecture that supports verbal actions would be embrained in a complex kinesthetic connectedness, and should not be considered as working independently of similarly organized cortico-cortical circuits (Falk, 2012). This suggests intimate connections between tongue imagined movements and verbal action that concern both the left and right hemispheres. The results of the current study provide support for the idea that imagined tongue movements, which are sophisticated kinesthetic systems in essence, could be the precursors of verbal actions.

## Data Availability Statement

The raw data supporting the conclusions will be made available by the authors, without undue reservation for all participants who allowed to make their data available.

## Ethics Statement

The studies involving human participants were reviewed and approved by Bond University Human Research Ethics Committee (BUHREC 16065).

## Author Contributions

IG and HM performed the study and prepared the manuscript. All authors contributed to the article and approved the submitted version.

## Conflict of Interest

The authors declare that the research was conducted in the absence of any commercial or financial relationships that could be construed as a potential conflict of interest.
